# A systematic review of mediation analysis frameworks in studies examining the determinants of cardiometabolic outcomes in people living with HIV

**DOI:** 10.1186/s12874-025-02498-1

**Published:** 2025-02-20

**Authors:** Peter Vanes Ebasone, Nasheeta Peer, Anastase Dzudie, Merveille Foaleng, Johney Melpsa, Andre Pascal Kengne

**Affiliations:** 1 Department of Medicine, University of Cape Town, Cape Town, South Africa; 2 Clinical Research Education Networking and Consultancy (CRENC), Yaounde, Cameroon; 3 Non-Communicable Disease Research Unit, South African Medical Research Council, Cape Town, South Africa; 4 Faculty of Medicine and Biomedical Sciences, University of Yaounde I, Yaounde, Cameroon; 5 Lown Scholars Program, Harvard T. H. Chan School of Public Health, Boston, USA

**Keywords:** HIV Infection, Antiretroviral Therapy, Cardiometabolic Diseases, Mediation Analysis, Epidemiological Research

## Abstract

**Introduction:**

Mediation analysis provides a more flexible mechanistic view of the causal relationship between HIV-related factors and cardiometabolic diseases. However, there is limited evidence on how mediation analysis is implemented in this specific research area. We aimed to describe the frameworks used in mediation analysis and examine how these analyses are conducted and reported in studies focusing on cardiometabolic outcomes among people living with HIV (PLWH).

**Methods:**

Following the PRISMA 2020 Guidelines, we comprehensively searched Medline, CINAHL, Africa-Wide Information and SCOPUS to identify observational studies that employed mediation analysis before October 2023. Two reviewers independently screened studies for eligibility. One reviewer performed data extraction, and two others reviewed the extracted information.

**Results:**

Nine studies met the inclusion criteria, predominantly focusing on the mediation effects of weight and obesity-related factors on the relationship between HIV serostatus, ART, and cardiometabolic outcomes. The review revealed a diverse application of both traditional and causal mediation frameworks. However, inconsistencies and gaps in reporting were noted, particularly in handling missing data, detailing identifiability assumptions, and the use of sensitivity analyses.

**Conclusion:**

While some studies of cardiometabolic risks among PLWH have embraced causal mediation frameworks, their overall application remains limited. In addition, we identified notable inconsistencies and gaps in reporting practices. To advance the field, researchers should not only integrate more rigorous causal mediation methods but also closely follow established reporting guidelines, such as the AGReMA Statement, to ensure greater transparency, reliability, and impact of future research.

**Supplementary Information:**

The online version contains supplementary material available at 10.1186/s12874-025-02498-1.

## Background

The intersection of HIV infection and cardiometabolic diseases presents a significant public health challenge [1]. With the increased use of antiretroviral therapies (ART), the life expectancy of people living with HIV (PLWH) has improved [2]. However, longevity in PLWH has led to a heightened risk of developing cardiometabolic diseases such as hypertension and diabetes mellitus [3, 4]. This heightened risk is attributed to traditional cardiometabolic risk factors, the chronic inflammatory effects of HIV infection and the direct effects of ART [5, 6]. Understanding the determinants of these risks and the underlying mechanisms is crucial for developing effective prevention and management strategies.

Mediation analysis offers a detailed view point to understand the causal relationships [7] between traditional, HIV related and psychosocial risk factors, and cardiometabolic diseases. While traditional causal analysis often focuses on the direct associations between these risk factors and cardiometabolic outcomes, mediation analysis goes deeper, exploring how and through what intermediate factors (mediators) these relationships occur. For example, it can elucidate the role of weight change or obesity, often influenced by HIV and ART, in the development of cardiometabolic diseases [8, 9]. Similarly, it can shed light on the role of inflammation, in mediating these health outcomes [10]. This approach provides a more comprehensive understanding of the causal mechanisms, revealing indirect pathways that might be overlooked in traditional analyses [7].

The importance of proper conduct and reporting in mediation analysis has been a recurring theme highlighted by researchers across various fields [11–14]. Despite the potential role of mediation analysis in understanding cardiometabolic risks among PLWH, a significant gap exists in how these analyses are properly conducted and reported [11]. Different mediation frameworks can yield varying results and are subject to distinct biases, assumptions, and challenges, including unmeasured confounding and measurement bias [15–18]. Despite the development of guidelines to ensure robust and transparent mediation analyses [19], it remains unclear whether these have been fully adopted in research on cardiometabolic outcomes in PLWH. Adherence to these guidelines clarifies assumptions, ensures proper adjustment for confounders, and promotes accurate interpretation of both direct and indirect effects [19]. Conversely, inadequate reporting of methodological choices and analytical steps can decrease the validity and reproducibility of findings, producing inconsistent evidence that potentially hinders the development of effective interventions.

This systematic review, therefore, aims to describe the frameworks used in mediation analysis and examine how these analyses are conducted and reported in studies focusing on cardiometabolic outcomes among PLWH. By evaluating the current state of research, the review highlights common pitfalls and suggests future improvements.

## Methods

This systematic review is reported in accordance with the Preferred Reporting Items for Systematic reviews and Meta-Analysis (PRISMA) 2020 Guidelines [20].

### Information sources and search strategy

We did a comprehensive search across MEDLINE via PubMed, CINAHL and Africa-Wide Information via EBSCO-HOST and SCOPUS to identify all relevant published studies. A predefined and sensitive search strategy was developed using combinations of MESH terms, CINAHL headings, and free words relating to cardiometabolic risk factors and diseases, mediation analysis and HIV/AIDS (Additional file 1). The last search was on 10^th^ October 2023.

### Screening and selection of studies

To be included in the review, studies had to; i) include PLWH, ii) report any of the following cardiometabolic risk factors and outcomes of interest: hypertension, systolic blood pressure (SBP), diastolic blood pressure (DBP), obesity, body mass index (BMI), waist circumference, triglycerides, high-density lipoprotein cholesterol (HDL-C), low-density lipoprotein cholesterol (LDL-C), total cholesterol, fasting blood sugar (FBS), random blood sugar (RBS), diabetes, stroke, atherosclerosis, ischaemic heart disease and sudden cardiac death, iii) be cross-sectional studies or cohort or case control studies that involved mediation analysis, published in English or French. We excluded case series, case reports, reviews, clinical trials, commentaries, and editorials, and studies that did not involve mediation analysis.

We used EPPI reviewer 4.0 [21] to screen titles and abstracts and full texts. One reviewer screened titles and abstracts of all studies, while another independently reviewed the titles and abstracts of a random third of studies retrieved from electronic searches. Two reviewers independently reviewed all studies included for full text screening. Disagreements were resolved through consensus and by consulting a third team member. The agreement between the reviewers was 95.6% for titles and abstracts and 90.3% for full text screening.

### Data extraction

Data was extracted using a purpose-design and piloted extraction form. One reviewer extracted data from all studies and two reviewers reviewed all extracted studies. Disagreements were resolved through consensus between the 3 reviewers. In addition to study and participant characteristics (first author name, year of publication, country, study design, mean or median age, sample size and study population), we extracted data on mediation frameworks and methodology and how the mediation analyses were reported.

### Mediation frameworks and methodology

**Fig. 1:**
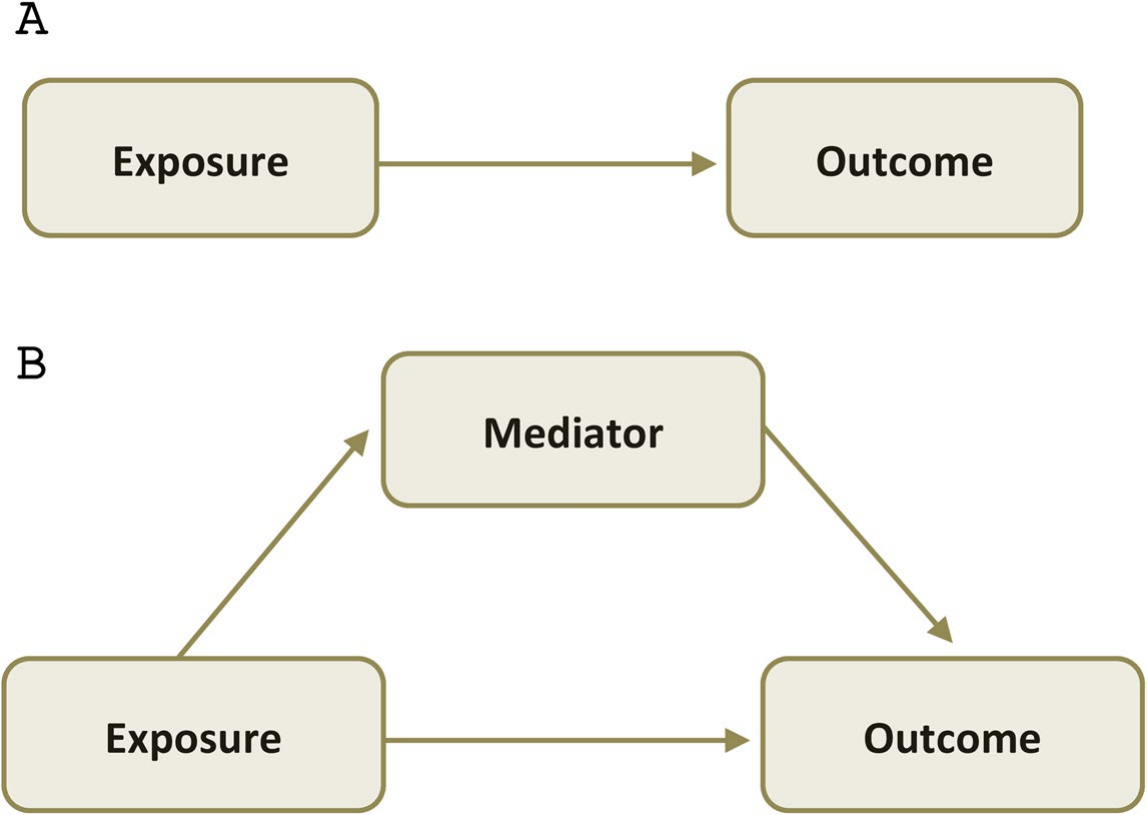
Diagram of a simple mediation model. Panel A of the graphic representation of the assumed causal model shows a direct link (path c) from 'Exposure' to 'Outcome,' depicting the total effect without mediators. Panel B introduces a 'Mediator,' creating two paths: 'Exposure' to 'Mediator' (path a) and 'Mediator' to 'Outcome,' (path b) illustrating the indirect effect. Additionally, the direct path from 'Exposure' to 'Outcome' (path c’) in Panel B indicates the direct effect, separate from the mediator’s influence

Typically, a simple mediator model involves three key components: the exposure (X), the mediator (M), and the outcome (Y) (Fig. 1). The mediator is the variable through which the exposure is hypothesized to exert its effect on the outcome [22]. The mediator(s) can be single, multiple, parallel, or serially placed between the exposure and outcome [13]. There are two primary frameworks for mediation analysis: traditional and causal mediation.

#### Traditional mediation analysis

Follows a path-analytic framework, often guided by the principles set out by Baron and Kenny in 1986 [22]. In this approach, the total effect of the exposure on the outcome is decomposed into direct (effect of X on Y not through M) and indirect (effect of X on Y through M) effects. Traditional mediation typically assumes linear relationships and often deals with a single mediator [7, 22]. The measures reported in this framework usually include coefficients representing these direct and indirect paths, often analysed through regression models. However, this approach may not adequately address issues such as non-linear relationships, multiple mediators, or complex causal pathways [17].

#### Causal mediation analysis

In contrast, incorporates concepts from causal inference, using counterfactuals to conceptualize what the results would be under different scenarios [17]. This framework is more flexible, allowing for the analysis of multiple mediators, non-linear relationships, and interaction effects [16, 17]. While both traditional and causal mediation approaches can adjust for measured confounders, causal mediation analysis typically makes the no-unmeasured-confounder assumptions explicit and provides formal tools—such as sensitivity analyses—to assess how unmeasured confounding might affect the estimates. By clearly stating these assumptions and offering avenues to evaluate their robustness, causal mediation methods often ensure greater rigor and transparency in addressing confounding variables [16]. Although in basic scenarios involving continuous mediators and outcomes modeled linearly, both traditional and causal mediation analyses yield similar effect estimates, causal mediation methods become more flexible and informative as complexity increases. Unlike traditional mediation analysis methods, causal mediation analysis accommodates the estimation of mediation effects for more complicated mediation. For example, they can handle non-linearities, time-varying mediators, and interactions more explicitly, and provide natural direct and indirect effects that retain their causal interpretation even in less straightforward settings [18]. This approach is particularly valuable in studies where the causal structure is complex or when the relationships between variables are not strictly linear [16–18].

The distinction between these frameworks is crucial as it influences the interpretation of mediation effects. Traditional mediation is often more straightforward and easier to implement but may oversimplify complex relationships. Causal mediation, while more complex, offers a more robust and adaptable understanding of the underlying mechanisms.

In our review, we extracted data on (i) the exposure(s), mediator(s), and outcome(s), (ii) number and nature of mediators, (iii) confounders, (iv) mediation framework used, (v) regression model employed, (vi) measures reported, and (vii) the software used. This information is essential to appreciate the methodological diversity and depth in the mediation analyses across the studies, providing insights into how different approaches can yield varying interpretations of the relationships among variables.

### Reporting of the mediation analysis

The Guideline for Reporting Mediation Analyses of Randomized Trials and Observational Studies (The AGReMA Statement) recommends that reporting of mediation analysis should be systematic and effective to ensure transparency, reproducibility, and accurate interpretation of research findings [19]. A comprehensive report should include (i) a graphic representation of the assumed causal model such as a path diagram or a Directed Acyclic Graph (DAG), which is vital for visually representing the assumed relationships among the exposure, mediator(s), and outcome. The path diagram clarifies the hypothesized pathways and makes the underlying assumptions of the mediation model explicit, aiding in the understanding of the causal framework [18]. It should also detail (ii) the handling of missing data within the study, including the techniques employed, such as multiple imputation or sensitivity analysis. This is crucial as the approach to missing data can significantly impact the results and interpretations of the mediation analysis, affecting the study's validity [19]. The (iii) rationale or motivation behind opting for mediation analysis should be clearly articulated, highlighting the theoretical or empirical basis suggesting a mediating relationship [7]. This justification is essential for understanding why mediation analysis is appropriate and what it aims to elucidate in the context of the study. Moreover, the report should include (iv) the specific conditions under which the mediation analysis was conducted, including model specifications and the nature of the variables involved. This information is critical for replicability and for other researchers to understand the applicability of the findings. Additionally, (v) detailing identifiability assumptions for the mediation effects, such as the absence of unmeasured confounding and the form of the relationships, is important for evaluating the robustness of the analysis [16]. Another critical aspect to report is (vi) the potential for interaction between the exposure and the mediator, indicating whether and how such interactions were tested or accounted for [16]. This is significant as it can influence the interpretation of the mediation effects and may reveal complex dynamics between the variables. Finally, (vii) the conduct and results of any sensitivity analysis should be included, assessing how robust the findings are to potential violations of the assumptions, such as unmeasured confounding [23]. This analysis is key to understanding the reliability and generalizability of the mediation effects.

We extracted data on whether the eligible studies reported these essential elements. This is fundamental for evaluating the methodological rigour and completeness of the mediation analysis conducted in the studies. It is also crucial for assessing the validity and generalizability of their findings.

## Results

### Summary of searches and study selection

**Fig. 2:**
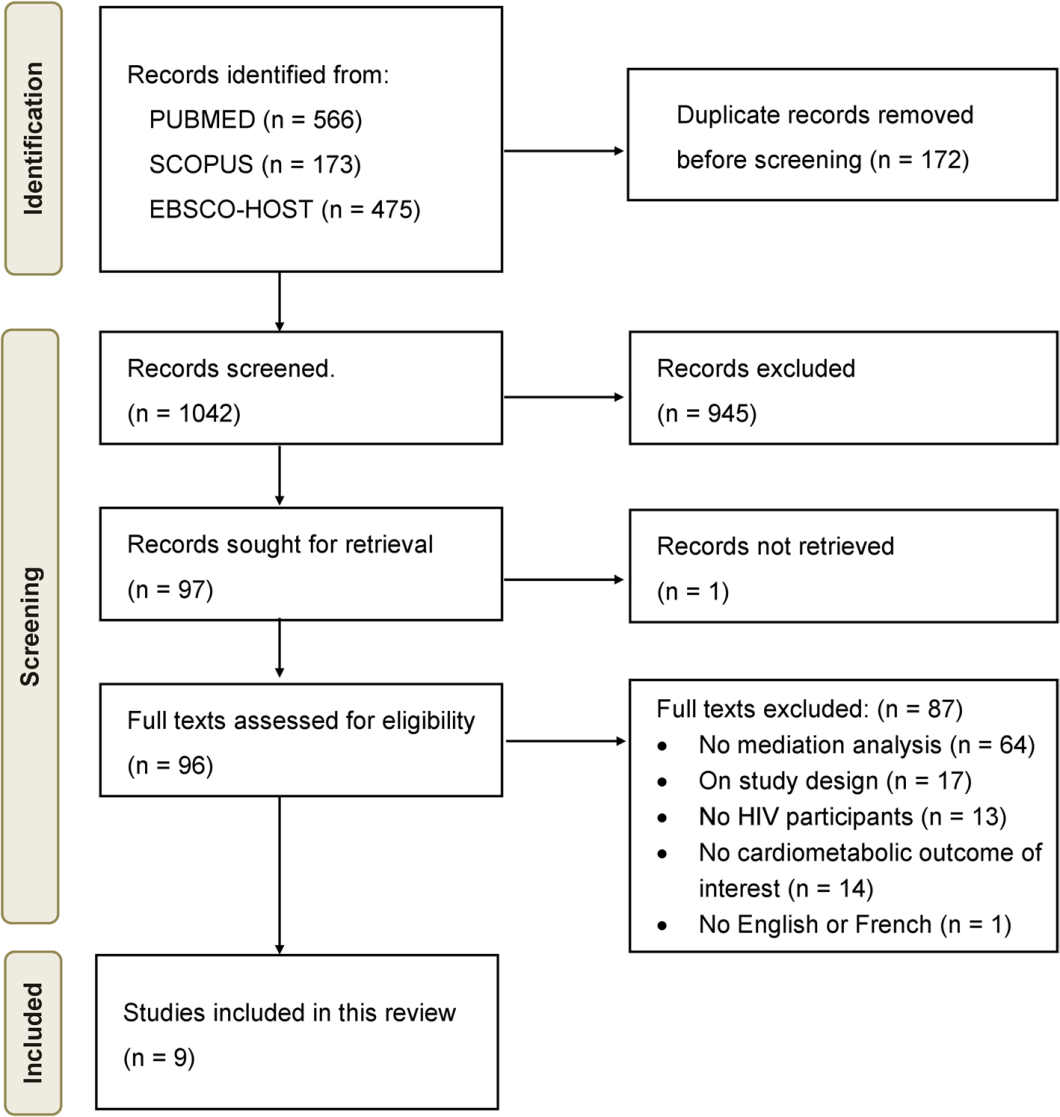
PRISMA flow diagram showing the selection process of studies included and excluded in the review

The study selection process is summarized in Fig. 2. In total, 1214 studies were identified via database searches. After deduplication, we screened the title and abstracts of 1042 articles, of which 96 were retrieved for full text screening. Of these, 9 articles met the inclusion criteria and were included in this review.

### Characteristics of included studies

Table 1 presents a summary of the individual studies included in this review. Of the 9 studies included, 6 were conducted in the United States, with one each in Nigeria, Uganda, and Italy. These studies, conducted between 1993 and 2019 and all published after 2016, mostly employed cohort study designs (6 studies), with the remainder being cross-sectional. A diverse array of cardiometabolic conditions was investigated, including blood pressure, hypertension, diabetes mellitus, atherosclerosis, ischaemic events, insulin resistance, and a composite of multiple conditions. Most studies (5 out of 9) considered mediation as a secondary analysis, while the remaining four treated it as a primary analysis.

**Table 1:** General description of studies included in the systematic review

Author (year)	Country	Study design	Participants age (years)	Mean or median age (years)	Study period	Sample size	Study population	Cardiometabolic outcome studied	Mediation is primary or secondary analysis?
Nduka (2016) [9]	Nigeria	Cross-sectional	≥ 18	37.6	August to November 2014	406	HIV-infected adult patients who were ART-naïve or exposed to HAART	Blood pressure	Primary
McIntosh (2017) [24]	United States	Cohort	21 to 62	41	1993 to 1997	61	HIV positive men and women partaking in a 10-week cognitive behavioural stress management intervention study	Blood pressure	Primary
Okello (2017) [25]	Uganda	Cross-sectional	≥ 40	45	June to October 2015	1115	HIV-infected and HIV-uninfected controls matched by age, sex, and neighbourhood	Blood pressure and hypertension	Secondary
Okello (2019) [8]	Uganda	Cohort	≥ 40	51.5	December 2013 to May 2018	309	HIV‐infected persons aged 40 and HIV‐uninfected controls who were gender‐ and age‐matched	Blood pressure	Secondary
Alcaide (2020) [10]	United States	Cross-sectional	18 to 50	36.15	December 2014 to June 2018	685	HIV infected and uninfected controls, cocaine, and non-cocaine users	Atherosclerosis	Primary
Li (2020) [26]	United States	Cohort	≥ 18		2003 to 2019	1781	HIV patients treated with atazanavir or darunavir	Ischaemic cardiac event or stroke	Secondary
Rebeiro (2021) [27]	United States and Canada	Cohort	≥ 18	41	January 2007 toDecember 2017	22,884	HIV infected cART-naive individuals in the NA-ACCORD cohort initiating their first regimen	Diabetes mellitus	Secondary
Milic (2022) [28]	Italy	Cohort	≥ 18	45	January 2004 to December 2019	2437	ART-experienced PWH, INSTI naive at baseline	Insulin resistance	Secondary
Friedman (2022) [29]	United States	Cohort	22 to 84	51.8	2008 to 2019	1806	sexual mcitatioinority men (SMM), 48.3% of participants of which were PWH	Composite including diabetes, hypertension and dyslipidaemia	Secondary

*HIV* Human Immunodeficiency Virus, *HAART* Highly Active Antiretroviral Therapy, *cART* Combination Antiretroviral Therapy, *PWH* People With HIV, *INSTI* Integrase Strand Transfer Inhibitor, *NA-ACCORD* North American AIDS Cohort Collaboration on Research and Design, *SMM* Sexual Minority Men, *CVD* Cardiovascular Disease, *ART* Antiretroviral Therapy

### Mediation frameworks and methodologies

Most studies (5 out of 9) assessed the effect of HIV serostatus and ART on cardiometabolic conditions like blood pressure, diabetes mellitus, and insulin resistance, with weight and obesity-related factors as key mediators. Okello et al., in two studies [8, 25] and Nduka et al. [9] both investigated the impact of HIV serostatus and ART, respectively, on blood pressure, with a focus on BMI and waist circumference as mediators. Similarly, Rebeiro et al. [27] and Milic et al. [28] explored the effects of initiating ART and switching to INSTI ART on diabetes mellitus and insulin resistance, respectively, considering weight change and BMI as key mediating factors. Overall, included studies considered different numbers of mediators, ranging from 1 to 7, and accounted for various confounders such as age, sex, smoking, and ethnicity.

Out of the 9 studies, 3 employed traditional mediation frameworks [9, 10, 24], 4 used causal mediation analysis, and 2 did not explicitly state if they used either of these two general frameworks [26, 27]. Among the studies utilizing traditional mediation frameworks, 2 were based on the Baron and Kenny method [9, 24], while a single study applied path analysis [10]. These traditional methods utilized linear regression models in 2 studies [9, 24] and structural equation modelling (SEM) in one study [10]. On the other hand, the studies adopting causal mediation analysis leveraged counterfactual approaches and parametric regression models to assess mediation effects [8, 25, 28]. The 2 studies that did not explicitly specify their mediation frameworks employed Cox proportional hazards regression, indicating a survival-based mediation analysis approach [26, 27]. In terms of the mediation effect types reported, traditional frameworks reported indirect effects [9, 10, 24], direct effects [9, 30], total effects [9, 10] and percentage mediated [9]. In the studies that used causal mediation frameworks [8, 25, 28, 29], a range of mediation effects were reported, including various forms of indirect and direct effects, total effects, and measures of percentage mediated. These effects, such as natural indirect effects, controlled direct effects, and the average causal mediation effects varied across the studies. A range of statistical software was used across the studies, including Mplus, SAS, Stata, and R (see Table 2).

**Table 2:** Summary of Mediation Frameworks and Methodologies in HIV-Related Studies

Author (Year)	Exposure(s)	Primary Outcome(s)	Mediator(s)	No. of Mediators	Confounders	Mediation Framework	Regression Models	Measures Reported	Analysis Software
Nduka (2016) [9]	HAART status (binary)	Blood pressure (continuous)	BMI, WC, BMI + WC (continuous)	3	Age, sex, smoking status, CD4 count, duration of HIV infection	Traditional: Baron and Kenny	Linear regression models	Indirect effect, direct effect, percentage mediated	Not specified
McIntosh (2017) [24]	9-month change in mood (continuous)	9-month blood pressure (continuous)	9-month change in urinary cortisol (continuous)	1	Baseline blood pressure (SBP and DBP)	Traditional: Baron and Kenny	Linear regressions	Direct effects, indirect effect	Mplus v6.12
Okello (2017) [25]	HIV serostatus (binary)	Systolic Blood Pressure (continuous)	BMI, Waist hip ratio	2	Age, sex, asset index, marital status, smoking, alcohol consumption, stress	Causal: Counterfactual approach	Linear and binary logistic regression	Indirect effect, direct effect, total effect, percentage mediated	Stata v13.0
Okello (2019) [8]	HIV serostatus (binary)	Blood pressure (continuous)	BMI, biomarkers of HIV inflammation and immune act (continuous)	4	Age, gender, smoking, physical activity	Causal: Parametric models	Linear regression models	Total effect, natural direct effect, total effect mediated effect	Stata v15
Alcaide (2020) [10]	HIV serostatus (binary)	Number of atherosclerotic plaques (continuous)	Inflammatory markers and MAP (continuous)	7	Age, BMI, smoking	Traditional: Path analysis	SEM	Indirect effect, total indirect effect	Mplus v8
Li (2020) [26]	ART (atazanavir) exposure (binary)	Ischemic cardiac event or stroke (binary)	Total bilirubin (continuous)	1	Hypertension, hyperlipidaemia, diabetes, smoking, sex, ethnicity, virologic failure, ritonavir use, age	Not specified	Cox proportional hazards regression	Not specified	SAS v9.4
Rebeiro (2021) [27]	cART regimen core class (categorical)	Diabetes Mellitus (binary)	Weight change (continuous)	1	Age, sex, race/ethnicity, HIV transmission, baseline weight, CD4 + count, HIV-1 RNA, cART initiation year	Not specified	Cox proportional hazards regression	Total and direct effect	R v3.4.4
Milic (2021) [28]	Switch to INSTI ART (binary)	Insulin resistance (binary)	% weight change, % BMI change (continuous)	2	Age, sex, baseline weight/BMI, HOMA-IR	Causal: Counterfactual approach	Cox proportional hazards regression	Average causal mediation effect, direct effect, total effect, percentage mediated	R v4.0.2
Friedman (2022) [29]	Black ethnoracial identity (binary)	Composite measure (continuous)	Experienced intersectional stigma (continuous)	1	Low-income status, Hispanic/Latinx ethnicity, bisexual behaviour, study site, age, HIV status	Causal: Poisson distribution, 4-way decomposition	Cross-sectional Poisson model, GLMM	Controlled direct effect, mediated interaction, natural direct effect, natural indirect effect, portion attributed to interaction, portion eliminated, pure indirect effect, total direct effect	SAS v9.4

*MAP* Mean Arterial Pressure, *SEM* Structural Equation Modelling, *BMI* Body Mass Index, *WC* Waist Circumference, *HAART* Highly Active Antiretroviral Therapy, *ART* Antiretroviral Therapy, *INSTI* Integrase Strand Transfer Inhibitor, *GLMM* Generalized Linear Mixed Models, *cART* Combination Antiretroviral Therapy, *HOMA-IR* Homeostatic Model Assessment for Insulin Resistance, *SBP and DBP* Systolic and Diastolic Blood Pressure, *HIV serostatus (binary)* Indicates whether a study participant is HIV-positive or HIV-negative, *Continuous and Binary Data* 'Continuous' refers to data that can take any value within a range, while 'Binary' refers to data with two categories, often represented as 0 or 1, *Mediation Frameworks* 'Traditional' refers to classical approaches like Baron and Kenny's method and path analysis, while 'Causal' refers to more recent approaches based on counterfactual reasoning, *Regression Models* Statistical methods used to estimate the relationships among variables, *Measures Reported* Types of statistical effects or outcomes reported in the study, *Analysis Software* Software tools used for statistical analysis in the study

### Reporting of the mediation analysis

**Table 3:** Reporting Characteristics of Mediation Analysis

Author (Year)	Path diagram Included?	Missing Data: Method	Power and sample size calculation for MA	Reason for MA	Conditions for MA	Confounder adjustment	Exposure-Mediator Interaction	Identifiability Assumptions	Sensitivity Analysis	Limitations
Nduka (2016) [9]	Yes	Yes: Not specified	No	Inform development of CVD interventions	No	Yes	No	No	No	Observational nature
McIntosh (2017) [24]	No	Yes: Complete case analysis	No	Improve understanding	No	Yes	No	No	No	Observational nature, unmeasured confounding
Okello (2017) [25]	Yes	No	No	Improve understanding	Yes	Yes	No	No	No	Unmeasured confounding
Okello (2019) [8]	Yes	No	Yes	Improve understanding	No	Yes	No	No	No	Observational nature, unmeasured confounding, recall bias
Alcaide (2020) [10]	Yes	Yes: Complete case analysis	No	Improve understanding	No	Yes	Yes	No	No	Unmeasured confounding, measurement error
Li (2020) [26]	No	Yes: Complete case analysis and Multiple imputation	No	Improve understanding	No	Yes	Yes	No	Yes	Unmeasured confounding
Rebeiro (2021) [27]	Yes	Yes: Not specified	No	Improve understanding	No	Yes	No	No	No	Observational nature, unmeasured confounding
Milic (2021) [28]	Yes	Yes: Not specified	No	Improve understanding	No	Yes	Yes	No	Yes	Unmeasured confounding, measurement error
Friedman (2022) [29]	No	Yes: Observed margins specification	No	Improve understanding	No	Yes	Yes	No	No	Recall and measurement bias

*MA*Mediation Analysis, *CVD* Cardiovascular Disease, *BP* Blood Pressure, *DBP* Diastolic Blood Pressure, *CORT* Cortisol, *HAART* Highly Active Antiretroviral Therapy, *BMI* Body Mass Index, *SBP* Systolic Blood Pressure, *INSTI* Integrase Strand Transfer Inhibitor, *DM* Diabetes Mellitus, *IR* Insulin Resistance, *EIS* Experienced Intersectional Stigma, *IMD* Mediated Interaction, *TAF* Tenofovir Alafenamide, *HCV* Hepatitis C Virus

Table 3 summarizes the reporting characteristics of mediation analysis in the included studies. Path diagrams were incorporated in 6 out of the 9 studies [8–10, 25, 28, 30]. With regards to missing data, 7 out of 9 studies acknowledged its presence, with 4 providing specific details on how it handled. Complete case analysis was used in 3 studies [25–27], multiple imputation in one [27], and observed margins specification was employed in another [29]. A single study reported power and sample size calculation for mediation analysis and included the formula [9]. The reason for conducting mediation analysis was universally alluded to, primarily to enhance understanding, with one study specifically aiming to develop interventions to decrease cardiovascular disease risk [10]. The study by McIntosh et al [24] was the only study that explicitly stated the conditions for mediation analysis. All studies mentioned confounder adjustment in at least one of the models. Exposure-mediator interactions were included in the models of 3 studies [8, 27, 29]. Notably, none of the studies explicitly detailed the identifiability assumptions required for their mediation analysis. Sensitivity analysis was reported in two studies [8, 27], but only one study provided details on the approach used [8]. All studies recognized limitations related to their mediation analysis, commonly citing the observational nature of the studies and the potential for unmeasured confounding.

## Discussion

This systematic review examined the frameworks, conduct, and reporting of mediation analyses among studies that predominantly focused on the mediation effects of weight and obesity-related factors on the relationship between HIV serostatus and ART and cardiometabolic conditions such as blood pressure, diabetes mellitus, and insulin resistance. Our findings illustrate the use of both traditional and causal mediation analysis frameworks and reveal notable inconsistencies and gaps in reporting. These findings highlight both progress and areas needing improvement in this research domain.

The ongoing debate in mediation analysis centres around choosing the most suitable approach, a decision influenced by a blend of statistical, theoretical, and practical considerations, including the researcher's experience and objectives [15]. Our review reveals a diverse application of both traditional and causal mediation frameworks, with some studies adopting causal mediation methods, especially in complex models like time-to-event outcomes. This adoption mirrors the broader movement in epidemiological research towards more adaptable, causally-oriented methods [31, 32]. Causal mediation analysis is generally preferred because it encourages researchers to explicitly examine and address the plausibility of the causal assumptions underlying their study, thereby fostering a more rigorous and transparent evaluation of effect estimates [16–18]. This approach is particularly advantageous in scenarios with complex relationships and unmeasured confounders. Even when there is some unmeasured confounding, most statistical packages for mediation analysis incorporate sensitivity analyses [16, 23]. These sensitivity analyses evaluate how effect estimates change under various assumptions about confounder influence, a capability often lacking in traditional frameworks [23, 33]. However, it's important to note that causal mediation analysis can be complex and requires a higher level of statistical expertise, which may pose challenges in interpretation and application [34]. Despite the availability of causal frameworks and analytical software since 2010, our reviewed studies using traditional methods were all conducted post-2015 when causal frameworks could have been adopted. This slow adoption of causal mediation analysis is reflected in a recent review of mediation analysis methods used in observational research, which showed the predominance of traditional mediation methods [35].

The types of mediation effects reported in our review also underscore the evolution in mediation analysis. Traditional frameworks typically assess direct, indirect, and total effects [7], essential for understanding the mediator's role between variables in simpler models. However, they usually assume linearity and might not adequately address complex causal relationships, especially with confounding or interaction effects. Causal mediation analysis, employing counterfactual approaches and advanced statistical models, offers a more refined understanding of these effects [16, 17, 36]. It allows for the estimation of natural direct and indirect effects, controlled direct effects, and interaction effects [16, 18], exemplified by studies like Friedman et al. in our review [29]. This depth is crucial for unravelling the complex factors influencing health outcomes in PLWH.

The reporting characteristics of mediation analysis in the studies we reviewed reveal several key trends and areas for improvement in this field. The notable inconsistencies and gaps in reporting in our review mirror findings in similar reviews of mediation analysis reporting [11–14, 35, 37, 38]. The use of path diagrams in 6 out of the 9 studies is a positive sign, indicating a rigorous approach to conceptualizing and communicating the assumed causal relationships in these studies. Graphic representations of the assumed causal model such as path diagrams are crucial for clarifying the causal pathways being tested and for identifying potential confounders and mediators. Their use in most of the studies suggests a growing recognition of the importance of transparent and well-structured causal reasoning in epidemiological research. However, the handling and reporting of missing data in these studies present a mixed picture. While it is commendable that 7 out of 9 studies acknowledged the presence of missing data, the methods used to address this issue varied. The predominant reliance on complete case analysis in 5 studies may raise concerns about potential biases, as this method assumes that the data are missing completely at random, which is often not the case in clinical and epidemiological research [39, 40]. The use of multiple imputation in one study represents a more robust approach to handling missing data, as it allows for the estimation of missing values based on observed data, thereby reducing potential biases [39]. Handling missing data is crucial but often challenging, requiring a proper understanding of the reasons and mechanisms behind the missingness, as well as the appropriate methods to address it [39]. It is recommended to plan for missing data even before data collection begins, using robust methods such as multiple imputation when appropriate, and conducting sensitivity analyses to assess the robustness of the findings [41, 42].

A notable gap in the reporting of studies reviewed was the lack of explicit details on the specific conditions and identifiability assumptions required for mediation analysis. This omission is significant because the validity of mediation analysis results heavily depends on these assumptions, such as the assumption of no unmeasured confounding between the mediator and the outcome [16, 18, 23]. Without clear reporting on these assumptions, it is challenging for readers to assess the robustness of the study findings [19]. The inclusion of exposure-mediator interactions in the models of 3 studies is a positive step, as it indicates an awareness of the potential for these interactions to influence the mediation process. However, the limited use of sensitivity analysis, reported in only two studies and detailed in just one, is a concern. Sensitivity analysis is crucial for assessing how robust the mediation effects are to potential violations of assumptions, such as the presence of unmeasured confounding [23, 33]. The limited reporting on this aspect suggests a need for more rigorous approaches to assessing and reporting the robustness of mediation analysis findings. Finally, the universal recognition of limitations related to mediation analysis in all studies, particularly concerning the observational nature of the data and the potential for unmeasured confounding, is an important aspect of transparent reporting. It reflects an awareness of the inherent challenges in establishing causal inferences from observational data. However, this acknowledgment also underscores the need for more advanced analytical techniques, such as those offered by causal mediation analysis frameworks, and for more rigorous and detailed reporting of the methods used to address these challenges.

Considering recent advancements in mediation analysis methods, future research, especially in areas like cardiometabolic risks among PLWH, should increasingly adopt causal mediation analysis frameworks. These frameworks not only offer deeper insights into underlying mechanisms but also enhance the validity and applicability of research findings [17, 35]. Researchers are thus encouraged to employ these advanced methods in their analyses to comprehensively capture the complex dynamics of the relationships they study. Furthermore, there is a need for the broad epidemiological research field to adopt current reporting guidelines for mediation analysis such as the AGReMA Statement [19], to enhance comparability and reproducibility of findings.

## Limitations and strengths

This systematic review, conducted in accordance with the PRISMA 2020 Guidelines, presents notable strengths, including a comprehensive search strategy across multiple databases and the inclusion of a diverse range of cardiometabolic outcomes, enhancing the breadth and applicability of its findings. Notably, this is the first review to specifically examine mediation analysis in cardiometabolic disorders among PLWH. However, the review also faces several limitations. The restriction to English and French publications might introduce language bias, potentially excluding relevant studies in other languages. The exclusion of certain study designs, such as clinical trials, may limit the comprehensiveness of the insights gained. Another limitation stems from the potential underreporting by authors due to word limitations in journals, which may restrict the space available to provide detailed methodological descriptions. This is particularly relevant for methods requiring meticulous implementation, as comprehensive reporting is essential to evaluate the appropriateness of their application. Moreover, the limited number of identified studies hinders our ability to fully appreciate the current landscape of mediation analysis application and reporting in this field. Since the use of mediation analysis in examining cardiometabolic outcomes among PLWH remains relatively new, the findings should be interpreted as an initial, exploratory overview rather than a definitive characterization. Nonetheless, by highlighting these early practices and gaps, we hope to stimulate interest, guide future research, and encourage the adoption of more robust methodologies and improved reporting standards moving forward.

## Conclusion

While some studies in our review adopted causal mediation frameworks, their overall use remains limited. Moreover, the reporting of mediation analyses frequently falls short of recommended standards, reducing both transparency and reproducibility. To advance the field, researchers should not only employ more rigorous causal mediation methods but also adhere closely to established reporting guidelines, such as the AGReMA Statement. Strengthening both methodological rigor and reporting practices will be essential to produce more reliable, interpretable, and ultimately actionable evidence in this important domain of epidemiological research.

## Supplementary Information

Additional file 1.

Additional file 2.

## Data Availability

All data generated or analysed during this study are included in this article.

## Abbreviations

AIDS: Acquired Immunodeficiency Syndrome
ART: Antiretroviral Therapies
BMI: Body Mass Index
DAG: Directed Acyclic Graph
DBP: Diastolic Blood Pressure
FBS: Fasting Blood Sugar
HDL-C: High-Density Lipoprotein Cholesterol
INSTI: Integrase Strand Transfer Inhibitor
LDL-C: Low-Density Lipoprotein Cholesterol
PLWH: People Living With HIV
PRISMA: Preferred Reporting Items for Systematic Reviews and Meta-Analysis
RBS: Random Blood Sugar
SBP: Systolic Blood Pressure
