# Supplementary material for: A systematic review of mediation analysis frameworks in studies examining the determinants of cardiometabolic outcomes in people living with HIV
Source: BMC Med Res Methodol. 2025 Feb 20;25:41. doi: 10.1186/s12874-025-02498-1 (PMC11844112; doi:10.1186/s12874-025-02498-1)
Supplement: Supplementary file 1 — Additional file 1. [file 12874_2025_2498_MOESM1_ESM.docx]

Title: **A systematic review of mediation analysis frameworks in studies examining the determinants of cardiometabolic outcomes in people living with HIV**

Databases: PubMed-MEDLINE, SCOPUS, CINAHL, Academic Search Premier, and Africa-Wide Information. The last search was on 10^th^ October 2023.

**PubMed-MEDLINE**

| **Search** | **Search Terms** |
| --- | --- |
| 1 | Cardiometabolic risk factors[mesh] OR Cardiometabolic risk factor*[tw] OR Cardiometabolic diseas*[tw] OR Cardiometabolic syndrome[tw] OR Cardiometabolic[tw] OR Cardiovascular[tw] OR Metabolic Syndrome[mesh] OR Metabolic Syndrome[tw] OR Reaven Syndrome[tw] OR Syndrome X[tw] OR Stroke[mesh] OR Stroke[tw] OR Cerebrovascular accident[tw] OR Transient Ischemic Attack[tw] OR Transient Ischaemic Attack[tw] OR Coronary Artery Disease[mesh] OR Ischemic heart disease[tw] OR Ischaemic heart disease[tw] OR myocardial infarction[tw] OR angina[tw] OR Death, Sudden, Cardiac[mesh] OR Sudden cardiac death[tw] OR SCD[tw] |
| 2 | Obesity[mesh] OR Obesity[tw] OR Obese[tw] OR Overweight[mesh] OR Overweight[tw] OR Adiposity[tw] OR Body mass index[tw] OR BMI[tw] OR Waist circumference[tw] OR hip circumference[tw] OR waist-to-hip ratio[tw] OR waist-to-height ratio[tw] |
| 3 | Hypertension[mesh] OR High blood pressure[tw] OR raised blood pressure[tw] OR Blood pressure[tw] OR systolic blood pressure[tw] OR diastolic blood pressure[tw] OR SBP[tw] OR DBP [tw] OR elevated blood pressure [tw] |
| 4 | Diabetes mellitus[mesh] OR diabetes[tw] OR diabetes mellitus[tw] OR type 2 diabetes mellitus[tw] OR type 2 diabetes[tw] OR hyperglycemia[tw] OR hyperglycaemia[tw] OR Glycated haemoglobin[tw] OR HbA1c[tw] OR Impaired fasting glucose[tw] OR Fasting glucose[tw] OR Random blood sugar[tw] OR dysglycemia[tw] OR dysglycaemia[tw] |
| 5 | Dyslipidemias[mesh] OR dyslipidaemia[tw] OR dyslipidemia[tw] OR lipid*[tw] OR cholesterol[tw] OR hyperlipidemia[tw] OR hyperlipidaemia[tw] OR hypercholesterolemia[tw] OR hypertriglyceridemia[tw] OR triglyceride*[tw] OR total cholesterol[tw] OR HDL[tw] OR LDL[tw] OR TG[tw] OR TC[tw] OR VLDL[tw] OR hyperlipoproteinemia[tw] OR Lipid disorder[tw] OR HDL cholesterol[tw] OR high-density lipoprotein cholesterol[tw] OR LDL cholesterol[tw] OR low-density lipoprotein cholesterol[tw] OR HDL-C[tw] OR LDL-C[tw] |
| 6 | Mediation analysis[mesh] OR Mediation analysis[tw] OR mediation[tw] OR mediation studies[tw] OR causal mediation[tw] OR causal[tw] OR mediator[tw] OR structural equation modelling[tw] OR Baron and Kenny[tw] OR MacKinnon[tw] OR product of coefficient[tw] OR sobel*[tw] OR process variable[tw] |
| 7 | HIV[mesh] OR HIV[tw] OR HIV/AIDS[tw] OR human immunodeficiency virus[tw] OR acquired immunodeficiency syndrome[tw] OR AIDS[tw] OR acquired immunodeficiency syndrome[mesh] OR HIV infection[tw] OR WHO stage[tw] OR Antiretroviral therapy, highly active[mesh] OR Antiretroviral therapy[tw] OR HAART[tw] OR ART[tw] OR CD4[tw] OR viral load[tw] |
| 8 | #1 OR #2 OR #3 OR #4 OR #5 |
| 9 | #6 AND #7 AND #8 |

**EBSCO-host: Africa-Wide Information and CINAHL**

| **Search** | **Search Terms** |
| --- | --- |
| S1 | TX cardiometabolic OR TX cardiovascular OR TX metabolic syndrome OR TX stroke OR TX cerebrovascular accident OR TX transient ischaemic attack OR TX coronary artery disease OR TX ischemic heart disease OR TX myocardial infarction OR TX angina OR TX sudden cardiac death |
| S2 | TX obesity OR TX overweight OR TX adiposity OR TX body mass index OR TX waist circumference OR TX waist-to-hip ratio |
| S3 | TX hypertension OR TX high blood pressure OR TX blood pressure OR TX systolic blood pressure OR TX diastolic blood pressure OR TX elevated blood pressure |
| S4 | TX diabetes Mellitus OR TX type 2 diabetes OR TX hyperglycemia OR TX glycated haemoglobin OR TX HbA1c OR TX fasting glucose OR TX dysglycemia |
| S5 | TX Hyperlipidemia OR TX lipids OR TX dyslipidemia OR TX cholesterol OR TX hypercholesterolemia OR TX triglycerides OR TX total cholesterol OR TX hdl cholesterol OR TX ldl cholesterol OR TX hyperlipidemia |
| S6 | S1 OR S2 OR S3 OR S4 OR S5 |
| S7 | TX mediation analysis OR TX mediation OR TX mediation studies OR TX causal mediation OR TX causal OR TX mediator OR TX structural equation modelling OR TX ( baron and kenny ) OR TX mackinnon OR TX product of coefficient OR TX sobel* OR TX process variables |
| S8 | TX hiv OR TX hiv/aids OR TX human immunodeficiency virus OR TX acquired immunodeficiency syndrome OR TX aids OR TX who stage OR TX antiretroviral therapy OR TX haart OR TX cd4 OR TX viral load |
| S9 | S6 AND S7 AND S8 |

**SCOPUS**

| **Search** | **Search Terms** |
| --- | --- |
| 1 | ( TITLE-ABS-KEY ( cardiometabolic )  OR  TITLE-ABS-KEY ( cardiovascular )  OR  TITLE-ABS-KEY ( metabolic  AND syndrome )  OR  TITLE-ABS-KEY ( stroke )  OR  TITLE-ABS-KEY ( cerebrovascular  AND accident )  OR  TITLE-ABS-KEY ( transient  AND ischaemic  AND attack )  OR  TITLE-ABS-KEY ( coronary  AND artery  AND disease )  OR  TITLE-ABS-KEY ( ischemic  AND heart  AND disease )  OR  TITLE-ABS-KEY ( myocardial  AND infarction )  OR  TITLE-ABS-KEY ( angina )  OR  TITLE-ABS-KEY ( sudden  AND cardiac  AND death ) ) |
| S2 | ( TITLE-ABS-KEY ( obesity )  OR  TITLE-ABS-KEY ( overweight )  OR  TITLE-ABS-KEY ( adiposity )  OR  TITLE-ABS-KEY ( body  AND mass  AND index )  OR  TITLE-ABS-KEY ( waist  AND circumference )  OR  TITLE-ABS-KEY ( waist-to-hip  AND ratio ) ) |
| S3 | ( TITLE-ABS-KEY ( hypertension )  OR  TITLE-ABS-KEY ( high  AND blood  AND pressure )  OR  TITLE-ABS-KEY ( blood  AND pressure )  OR  TITLE-ABS-KEY ( systolic  AND blood  AND pressure )  OR  TITLE-ABS-KEY ( diastolic  AND blood  AND pressure )  OR  TITLE-ABS-KEY ( elevated  AND blood  AND pressure ) ) |
| S4 | ( TITLE-ABS-KEY ( diabetes  AND mellitus )  OR  TITLE-ABS-KEY ( type  2  diabetes )  OR  TITLE-ABS-KEY ( hyperglycemia )  OR  TITLE-ABS-KEY ( glycated  AND haemoglobin )  OR  TITLE-ABS-KEY ( hba1c )  OR  TITLE-ABS-KEY ( fasting  AND glucose )  OR  TITLE-ABS-KEY ( dysglycemia ) ) |
| S5 | ( TITLE-ABS-KEY ( hyperlipidemia )  OR  TITLE-ABS-KEY ( lipids )  OR  TITLE-ABS-KEY ( dyslipidemia )  OR  TITLE-ABS-KEY ( cholesterol )  OR  TITLE-ABS-KEY ( hypercholesterolemia )  OR  TITLE-ABS-KEY ( triglycerides )  OR  TITLE-ABS-KEY ( total  AND cholesterol )  OR  TITLE-ABS-KEY ( hdl  AND cholesterol )  OR  TITLE-ABS-KEY ( ldl  AND cholesterol )  OR  TITLE-ABS-KEY ( hyperlipidemia ) ) |
| S6 | #1 OR #2 OR #3 OR #4 OR #5 |
| S7 | ( TITLE-ABS-KEY ( mediation  AND  analysis )  OR  TITLE-ABS-KEY ( mediation  AND  studies )  OR  TITLE-ABS-KEY ( causal  AND  mediation )  OR  TITLE-ABS-KEY ( mackinnon )  OR  TITLE-ABS-KEY ( structural  AND  equation  AND  modelling )  OR  TITLE-ABS-KEY ( baron  AND  kenny )  OR  TITLE-ABS-KEY ( product  AND  of  AND  coefficient )  OR  TITLE-ABS-KEY ( sobel* )  OR  TITLE-ABS-KEY ( mediation ) ) |
| S8 | ( TITLE-ABS-KEY ( hiv )  OR  TITLE-ABS-KEY ( hiv/aids )  OR  TITLE-ABS-KEY ( human  AND immunodeficiency  AND virus )  OR  TITLE-ABS-KEY ( acquired  AND immunodeficiency  AND syndrome )  OR  TITLE-ABS-KEY ( aids )  OR  TITLE-ABS-KEY ( who  AND stage )  OR  TITLE-ABS-KEY ( antiretroviral  AND therapy )  OR  TITLE-ABS-KEY ( haart )  OR  TITLE-ABS-KEY ( cd4 )  OR  TITLE-ABS-KEY ( viral  AND load ) ) |
| S9 | S6 AND S7 AND S8 |

**Combined SCOPUS search strategy**

( ( ( TITLE-ABS-KEY ( cardiometabolic )  OR  TITLE-ABS-KEY ( cardiovascular )  OR  TITLE-ABS-KEY ( metabolic  AND syndrome )  OR  TITLE-ABS-KEY ( stroke )  OR  TITLE-ABS-KEY ( cerebrovascular  AND accident )  OR  TITLE-ABS-KEY ( transient  AND ischaemic  AND attack )  OR  TITLE-ABS-KEY ( coronary  AND artery  AND disease )  OR  TITLE-ABS-KEY ( ischemic  AND heart  AND disease )  OR  TITLE-ABS-KEY ( myocardial  AND infarction )  OR  TITLE-ABS-KEY ( angina )  OR  TITLE-ABS-KEY ( sudden  AND cardiac  AND death ) ) )  OR  ( ( TITLE-ABS-KEY ( obesity )  OR  TITLE-ABS-KEY ( overweight )  OR  TITLE-ABS-KEY ( adiposity )  OR  TITLE-ABS-KEY ( body  AND mass  AND index )  OR  TITLE-ABS-KEY ( waist  AND circumference )  OR  TITLE-ABS-KEY ( waist-to-hip  AND ratio ) ) )  OR  ( ( TITLE-ABS-KEY ( hypertension )  OR  TITLE-ABS-KEY ( high  AND blood  AND pressure )  OR  TITLE-ABS-KEY ( blood  AND pressure )  OR  TITLE-ABS-KEY ( systolic  AND blood  AND pressure )  OR  TITLE-ABS-KEY ( diastolic  AND blood  AND pressure )  OR  TITLE-ABS-KEY ( elevated  AND blood  AND pressure ) ) )  OR  ( ( TITLE-ABS-KEY ( diabetes  AND mellitus )  OR  TITLE-ABS-KEY ( type  2  diabetes )  OR  TITLE-ABS-KEY ( hyperglycemia )  OR  TITLE-ABS-KEY ( glycated  AND haemoglobin )  OR  TITLE-ABS-KEY ( hba1c )  OR  TITLE-ABS-KEY ( fasting  AND glucose )  OR  TITLE-ABS-KEY ( dysglycemia ) ) )  OR  ( ( TITLE-ABS-KEY ( hyperlipidemia )  OR  TITLE-ABS-KEY ( lipids )  OR  TITLE-ABS-KEY ( dyslipidemia )  OR  TITLE-ABS-KEY ( cholesterol )  OR  TITLE-ABS-KEY ( hypercholesterolemia )  OR  TITLE-ABS-KEY ( triglycerides )  OR  TITLE-ABS-KEY ( total  AND cholesterol )  OR  TITLE-ABS-KEY ( hdl  AND cholesterol )  OR  TITLE-ABS-KEY ( ldl  AND cholesterol )  OR  TITLE-ABS-KEY ( hyperlipidemia ) ) ) )  AND  ( ( TITLE-ABS-KEY ( hiv )  OR  TITLE-ABS-KEY ( hiv/aids )  OR  TITLE-ABS-KEY ( human  AND immunodeficiency  AND virus )  OR  TITLE-ABS-KEY ( acquired  AND immunodeficiency  AND syndrome )  OR  TITLE-ABS-KEY ( aids )  OR  TITLE-ABS-KEY ( who  AND stage )  OR  TITLE-ABS-KEY ( antiretroviral  AND therapy )  OR  TITLE-ABS-KEY ( haart )  OR  TITLE-ABS-KEY ( cd4 )  OR  TITLE-ABS-KEY ( viral  AND load ) ) )  AND  ( ( TITLE-ABS-KEY ( mediation  AND  analysis )  OR  TITLE-ABS-KEY ( mediation  AND  studies )  OR  TITLE-ABS-KEY ( causal  AND  mediation )  OR  TITLE-ABS-KEY ( mackinnon )  OR  TITLE-ABS-KEY ( structural  AND  equation  AND  modelling )  OR  TITLE-ABS-KEY ( baron  AND  kenny )  OR  TITLE-ABS-KEY ( product  AND  of  AND  coefficient )  OR  TITLE-ABS-KEY ( sobel* )  OR  TITLE-ABS-KEY ( mediation ) ) )
